# Supplementary material for: A set of multi-entry identification keys to African frugivorous flies (Diptera, Tephritidae)
Source: Zookeys. 2014 Jul 24;(428):97–108. doi: 10.3897/zookeys.428.7366 (PMC4143993; doi:10.3897/zookeys.428.7366)
Supplement: Supplementary material 4 — Key to Capparimyia [file zookeys-428-097-s004.zip › SF4_ZooKeys_key to Capparimyia/lucid_player/help/help/registration_and_activation.htm]

Registering the Lucid Player (Application Edition)


# Registering the Lucid Player (Application Edition)

If you have purchased a key containing the Application Edition of the
Lucid3 Player, you may register your Player in order to receive notification
of updates. To register, click the *Register* button on the splash
screen.

**Note**
that if you have purchased and activated a copy of the Lucid3 Builder,
you do not need to register your Lucid3 Player.

On clicking *Register*, the following dialog box will be displayed:

Follow the three steps outlined in the activation dialog.

They are:

**Step 1**- Copy *all* of the Registration
Code text, then click the ‘Get Activation Code’ button. Your default web
browser should then open the Lucidcentral.org web
page.

**Step 2** -  If
you are a member of Lucidcentral, login. If you are
not yet a member, register with Lucidcentral by clicking
Join Lucidcentral.

After logging in or registering you will then be automatically directed
to the activation page. If not, the direct address is: http://www.lucidcentral.org/activation/

If you are registered but cannot remember your password at the login
page, click the *E-mail me my password* link.

**Ensure** you select
the correct product you wish to activate in the Step 1 dropdown box. To
activate your new Lucid3 player, choose '**Lucid Player v3.3 or greater**'.

Next, paste the copied registration code
into the web page text box located at Step 2, as shown below.

Next, click the ‘**Activate**’ button.
You should see the following page:

**Step 3**-
Copy *all* of the activation code from the text box and paste it
into the *Activation Code* text box on the Lucid Activation dialog
(shown below). Click the *Register* button to complete the process.

## Activation Notes for Network Administrators

To make the activation process more flexible
for computers that are centrally administered and have restricted privileges
for non-administrators, the default location for storing the activation
files is the user’s default home folder, where a ‘.lucid’ folder will
be created.

For example  
In the Windows XP operating system, the activation files will be stored
at:  
C:\Documents and Settings\<username>\.lucid.

If desired, registration and activation
files can also be placed into the Lucid installation folder. This will
allow more than one user to use Lucid on the same computer. Alternatively,
you may copy the ‘.lucid’ folder into other users’ home folders.
